# Chloroplast genome resources and molecular markers differentiate rubber dandelion species from weedy relatives

**DOI:** 10.1186/s12870-016-0967-1

**Published:** 2017-02-02

**Authors:** Yingxiao Zhang, Brian J. Iaffaldano, Xiaofeng Zhuang, John Cardina, Katrina Cornish

**Affiliations:** Department of Horticulture and Crop Science, The Ohio State University, Ohio Agricultural Research and Development Center, 1680 Madison Avenue, Wooster, OH 44691 USA

**Keywords:** Chloroplast genome, Rubber, Species-specific single nucleotide polymorphism markers, *Taraxacum brevicorniculatum*, *Taraxacum kok-saghyz*, *Taraxacum officinale*

## Abstract

**Background:**

Rubber dandelion (*Taraxacum kok-saghyz*, TK) is being developed as a domestic source of natural rubber to meet increasing global demand. However, the domestication of TK is complicated by its colocation with two weedy dandelion species, *Taraxacum brevicorniculatum* (TB) and the common dandelion (*Taraxacum officinale*, TO). TB is often present as a seed contaminant within TK accessions, while TO is a pandemic weed, which may have the potential to hybridize with TK. To discriminate these species at the molecular level, and facilitate gene flow studies between the potential rubber crop, TK, and its weedy relatives, we generated genomic and marker resources for these three dandelion species.

**Results:**

Complete chloroplast genome sequences of TK (151,338 bp), TO (151,299 bp), and TB (151,282 bp) were obtained using the Illumina GAII and MiSeq platforms. Chloroplast sequences were analyzed and annotated for all the three species. Phylogenetic analysis within Asteraceae showed that TK has a closer genetic distance to TB than to TO and *Taraxacum* species were most closely related to lettuce (*Lactuca sativa*). By sequencing multiple genotypes for each species and testing variants using gel-based methods, four chloroplast Single Nucleotide Polymorphism (SNP) variants were found to be fixed between TK and TO in large populations, and between TB and TO. Additionally, Expressed Sequence Tag (EST) resources developed for TO and TK permitted the identification of five nuclear species-specific SNP markers.

**Conclusions:**

The availability of chloroplast genomes of these three dandelion species, as well as chloroplast and nuclear molecular markers, will provide a powerful genetic resource for germplasm differentiation and purification, and the study of potential gene flow among *Taraxacum* species.

**Electronic supplementary material:**

The online version of this article (doi:10.1186/s12870-016-0967-1) contains supplementary material, which is available to authorized users.

## Background

Rubber dandelion (*Taraxacum kok-saghyz* Rodin, TK) is being developed as an alternative natural rubber source in response to increasing global demand and instability of current sources. Natural rubber production is fragile due to its reliance on a single source, the Brazilian or Para rubber tree (*Hevea brasiliensis* Muell. Arg.), which is cultivated as clones mostly in Southeast Asia [1]. This production could be easily disrupted by the introduction of South American Leaf Blight, a fatal fungal disease caused by *Microcyclus ulei* [2], which is currently controlled by quarantine measures. Moreover, *Hevea* rubber production is also threatened by high labor costs, due to the necessity of tapping latex from the trees by hand, and land competition with palm plantations [1]. To establish a more sustainable and mechanized natural rubber production system, TK has been explored in many temperate countries as a potential domestic rubber-producing crop [3].

TK, which originated in southeastern Kazakhstan as a wild plant [4], is a diploid (2x = 16) outcrossing, self-incompatible species. TK was cultivated extensively in the Union of Soviet Socialist Republics (USSR) and the US throughout the 1930s and during World War II to help alleviate wartime-induced natural rubber shortages [5]. At that time, rubber yields for TK were reported between 150 and 500 kg ha^−1^ y^−1^ [5]. Higher rubber production potential of TK has recently been demonstrated in studies where germplasm with a rubber content of 5–6% of root dry weight was grown in outdoor planting boxes to yield the equivalent of 1300 kg ha^−1^ in a 6-month period (Cornish, unpublished), which is comparable to the yield of rubber tree (500–3000 kg ha^−1^ y^−1^) [1]. Reaching comparable yields in large scale field production is a challenging endeavor, but coupled with germplasm with much higher rubber concentrations (up to 30%), commercially viable yields appear achievable [5]. Moreover, its wide environmental adaptation and fast generation time make TK one of the most promising alternate rubber producing plants. Rubber production from TK is expected to reduce the need to import rubber, mitigate production shortfalls, stabilize global rubber prices, as well as ensure rubber supplies should rubber tree production be threatened.

The domestication of TK is complicated by two additional dandelion species, *Taraxacum brevicorniculatum* Koroleva (TB) and *Taraxacum officinale* F.H. Wigg. (TO, common dandelion). TK, TB, and TO are sympatric species, and germplasm collections are often mixed [3]. TB is a triploid (3x = 24), which exhibits obligate apomixis, where clonal seeds are produced without pollination. TB also produces natural rubber in its roots, albeit to a lesser extent than TK (approximately 2–3% of the dry weight in TB, compared to as high as 30% in TK) [5, 6]. However, TB is a more vigorous species than TK with a high accumulation of biomass similar to TO. Recent molecular biology studies have used TB to investigate functions of genes related to rubber biosynthesis [6–8]. TB and TK share the same geographical origin and have been co-introduced into North America and Europe, where TB is often an unintentional seed contaminant. Therefore, TB has often been misidentified as TK in many *ex situ* germplasm collections until TB and TK were discriminated using taxonomic and Amplified Fragment Length Polymorphism (AFLP) analyses [3].

TO, the ubiquitous weedy dandelion, is distributed worldwide and can be found in all states and provinces of the United States and Canada, respectively [9]. TO has virtually no rubber production, although it does produces a milky latex, and is a vigorous, highly successful weed. TO is a perennial and is most successful as an agricultural weed in pastures and no-till systems. All TO reported in North America are obligate apomictic triploids (3x = 24) [10, 11]. However, sexual, diploid TO (2x = 16) has been identified in Europe [12].

TK domestication would involve large plantings and possibly the introduction of genetic modifications to improve agronomic performance and rubber yield. The potential for TK and TO to hybridize raises concerns about gene flow between species. There are two potential pathways of gene flow: pollen-mediated gene flow and seed-mediated gene flow [13]. In pollen-mediated gene flow, transgenes contained in TK pollen could potentially be introduced into TO and produce hybrid progeny with novel traits. Alternatively, TK could potentially serve as the pollen acceptor and be fertilized by TO pollen to produce hybrid progeny with weedy traits. In the case of seed-mediated gene flow, progeny produced by TK could be from TK x TK crosses, interspecific hybridization, or through the “mentor effect”, where self-incompatibility is broken down by the introduction of polyploid pollen [14, 15]. Similarly, in the case of pollen mediated gene flow, apomictically produced TO seeds would inevitably be mixed with the seeds of potential hybrids. In order to understand the potential for gene flow between TK and TO, species-specific molecular markers are needed to differentiate interspecific hybrids from apomictically produced TO and self-pollinated TK.

Considering the differences in rubber content and reproduction system among the aforementioned three species, as well as the potential production of TK/TO hybrids, it is important to distinguish them in studies related to molecular genetics, genomics, plant breeding, and gene flow risk assessment. Since the fecundity of weedy dandelions (TB and TO) has been reported to be 40 times higher than TK, once seeds from weedy dandelion are introduced into TK fields, the contamination can be magnified significantly through a single generation [5]. Information that can be used to resolve three dandelion species, as well as their potential hybrids, includes data on morphology and ploidy. However, morphological data may vary through developmental stage and is highly reliant on the experience of the observers. Ploidy detection using flow cytometry cannot be easily multiplexed and has a high cost of entry. Moreover, morphology and genome size of a potential hybrid may overlap with those of the three dandelion species. Therefore, it is necessary to develop molecular markers to provide an accurate and high throughput approach for species and hybrid differentiation.

One source of sequence diversity that can be used to differentiate species is the chloroplast genome. Due to the slower evolution of chloroplast genomes compared to nuclear genomes, chloroplast sequences have often been used for phylogenetic studies and species identification [16, 17]. Therefore, the development of chloroplast markers will provide an accurate molecular tool to differentiate *Taraxacum* species. Moreover, the genetic information in angiosperm chloroplasts is inherited maternally in most cases, making the chloroplast genome a good indicator of maternal ancestry [18]. The maternal parent could be easily identified in putative hybrid progeny in the absence of parental information, regardless of how many generations have past.

In previous studies, barcoding regions generated from chloroplast sequences have been used for phylogenetic analysis and species differentiation [16, 19]. However, the application of barcoding regions involves re-sequencing those regions of the tested plant samples. Chloroplast Single Nucleotide Polymorphism (SNP) markers were developed in recent studies due to their greater abundance in the genome and better resolution of populations [20, 21]. Since SNP detection can be easily multiplexed and applied to large populations, species differentiation using SNP markers is more practical and conducive to larger experiments.

To identify hybrids, chloroplast markers alone are insufficient, as they are dominant and only indicate maternal ancestry; however, chloroplast markers may be complemented with markers from the nuclear genomes of these species. To date only limited genomic resources are available for TK and TO; 16,441 expressed sequence tags (ESTs) derived from TK root RNA can be found on the National Center for Biotechnology Information (NCBI) (Collins J, Whalen MC, Nural-Taban AH, Scott D, Hathwaik U, Lazo GR, Cox K, Durant K, Woolsey R, Schegg K, et al. Genomic and proteomic identification of candidates genes and proteins for rubber biosynthesis in *Taraxacum kok-saghyz* (Russian dandelion). 2009. Unpublished; Shintani D. Using EST from *Taraxacum kok-saghyz* root cDNA library to generate candidate rubber biosynthetic genes. 2005. Unpublished). More EST data obtained from whole plants (41,294 ESTs, 16,858 unigenes) are available for TO [22, 23]. No TB sequence data have been reported.

In this study, chloroplast genomes have been sequenced for TK, TB and TO and chloroplast markers have been developed and validated. At the same time, nuclear markers were developed using previously published ESTs. The genomic and marker resources described in this paper will not only provide a molecular toolkit for germplasm identification and purification, but also allow accurate gene flow studies between TK and TO.

## Methods

### Chloroplast genome sequencing

To generate a complete TK chloroplast genome sequence, chloroplast DNA was extracted from a mixture of genetically distinct TK plants. To reduce polysaccharide content, which interferes with DNA extraction, young leaves were harvested from 1 to 2 month-old greenhouse grown TK plants subjected to a 2-day dark treatment before harvesting. About 20 g leaf tissue were ground in liquid nitrogen and suspended in 400 ml grinding buffer (0.35 M sorbitol, 50 mM HEPES/KOH, pH 7.5, 2 mM EDTA, 1 mM MgCl_2_, 1 mM MnCl_2_ and 4.4 mM sodium ascorbate (added just before use) (modified from [24, 25]). After filtering the tissue through four layers of miracloth, the filtrate was collected by centrifuging at 4500 × g for 20 min. The re-suspended pellets were placed on the top of a 30–50% sucrose gradient and centrifuged for 45 min at 10,000 × g, at 4 °C, in a swinging bucket rotor. The intact chloroplasts formed a layer between the 30 and 50% sucrose and were separated from the broken chloroplast remnants. Isolated chloroplasts were treated by DNase using Ambion® TURBO DNA-free™ Kit (Thermo Fisher Scientific Inc., Waltham, MA, USA) to degrade nuclear DNA. Chloroplast DNA was extracted using GenElute™ Plant Genomic DNA Miniprep kit (Sigma-Aldrich®, St. Louis, MO, USA) and enriched using the REPLI-g® Mini Kit (Qiagen, Inc., Hilden, Germany). DNA quality was initially checked and quantified using a NanoDrop® ND-1000 Spectrophotometer (NanoDrop Technologies, Inc., Wilmington, DE, USA). Distinctive individual band patterns shown after DNA digestion by restriction enzyme *EcoR*I indicated the high percentage of chloroplast DNA. DNA was submitted to The Molecular and Cellular Imaging Center (MCIC) at the Ohio Agricultural Research and Development Center (OARDC) for additional quality control and sequencing using the Illumina GAII sequencing platform.

To generate TK chloroplast genomes from multiple genotypes as well as complete TO and TB chloroplast genomes, three species were sequenced by MiSeq. A total of 24 genotypes were selected for TK, including 19 USDA lines, three mixed genotypes from USDA lines and a single cytoplasmic male sterile line (Additional file 1). All the USDA lines used in this study were obtained from the USDA-ARS National Plant Germplasm System (NPGS). These samples were collected in southeast Kazakhstan in 2008, from an area delineated by 42.79949 N to 43.06724 N, and 79.17952E to 80.08643E [26]. Detailed information of this collection can be obtained through the NPGS database, Germplasm Resources Information Network (GRIN) at http://www.ars-grin.gov/npgs/ [27]. Additional plants were selected from individual crosses between plants of specific USDA Accessions. All of the genotypes we selected to represent TK were self-incompatible and outcrossing, without variance in genome size. Twenty-four TO genotypes from a global collection of TO seed, including seed collected from North America, Europe and China, were used for sequencing (Additional file 2). All TO seeds used in this study were donated by weed scientists and other collaborators voluntarily, and collected by Prof. John Cardina (Ohio Agricultural Research and Development Center, The Ohio State University, Wooster, OH, USA). No permissions were required to obtain these seeds. TO seeds were identified based on the plant morphology and reproductive system. A TB “Clone A” donated by Peter van Dijk (Keygene, Wageningen, Netherlands), which originally came from the Botanical garden, Marburg, Germany, as well as 11 genotypes descended from plants collected from Kazakhstan and distributed broadly by Dr. Anvar Buranov (Nova-BioRubber Green Technologies Inc., Canada) were used for TB chloroplast sequencing (Additional file 3) [3]. All TO and TB plants used produced full seed set without pollination and exhibited apomixis after emasculation, with the exception of a single diploid, sexual TO accession, which was deliberately included. The total DNA from 60 leaf samples was extracted using a 2% cetyl trimethylammonium bromide (CTAB) DNA extraction protocol [28]. DNA amount was normalized to 1 ng μL^−1^ and used for entire chloroplast genome amplification by Long Range Polymerase Chain Reaction (PCR) using Q5® High-Fidelity DNA Polymerase (New England Biolabs Inc., Ipswich, MA, USA). Primers were designed on the conserved regions of the draft TK chloroplast sequence generated by the Illumina GAII data (Additional file 4). Amplified fragments were normalized within each species to have the same molarity and submitted for MiSeq sequencing. The 24 genotypes of TK, 24 genotypes of TO and 12 genotypes of TB were sequenced in a single MiSeq run. A library was made for each species, which was tagged using different barcoding sequences to separate short reads for each species. Individual accessions were not tagged separately.

### Chloroplast genome assembly and annotation

Paired-end reads were generated for multiple genotypes of TK, TO, and TB by the Illumina GAII and MiSeq sequencing platforms. Quality control was conducted using the FASTX-Toolkit [29]. For TK GAII data, the quality cutoff score was 40 (-q). A quality score of 20 was used for all Miseq data. By using the assembly program Velvet (version 1.2.10), with parameters, kmer = 35, -cov_cutoff = 20, a complete TK chloroplast genome sequence was generated from high quality GAII short reads [30]. Three contigs sized at 18,568, 24,353 and 84,064 bp long were generated. The 18,568 and 84,064 bp contigs had coverages of 344 and 343, respectively, representing the single copy regions. The 24,353 bp contig had a higher coverage of 834, as there are two copies of this region in a chloroplast haplotype. No Ns were included in the contigs. TO and TB short reads were assembled using the same method mentioned above with the quality score of 20. Assembled contigs were further mapped to the TK chloroplast genome as a reference by BLASTn to generate the entire chloroplast genomes [31].

Complete chloroplast genomes of TK, TO, and TB were annotated using the Dual Organellar GenoMe Annotator (DOGMA) [32]. Annotation errors were manually corrected. An annotation map was generated using OrganellarGenomeDRAW (OGDRAW) [33].

### Phylogenetic analysis in the Asteraceae and comparative analysis within *Taraxacum* genus

Phylogenetic analysis was conducted using the Rubisco (Ribulose-1, 5-bisphosphate carboxylase/oxygenase) large subunit gene *rbc*L from TK, TO, TB and other 27 species in the Asteraceae with available chloroplast genome sequences (Additional file 5). Multiple sequence alignments were carried out using ClustalW, followed by phylogenetic tree generation using MEGA6 [34]. The Maximum Likelihood method was used and the tree with the highest log likelihood was obtained [35].

To analyze the similarities and divergences of the TK, TO, and TB chloroplast genomes, complete chloroplast sequences of these three species were input into the mVISTA program, along with their annotation information [36, 37]. The Shuffle-LAGAN mode was chosen for comparative analysis [38]. The TK chloroplast sequence was used as the reference genome.

### Chloroplast species-specific marker discovery

To develop chloroplast species-specific markers between TK and TO, TO short reads were mapped to the TK chloroplast genome sequence using Bowtie 2 [39]. Variants between TK and TO were detected by Freebayes using the default parameters [40]. TK short reads were further mapped to the TK chloroplast genome to eliminate variants which were not fixed within TK. Variants between TK and TO, but fixed within each species, were considered candidate species-specific markers.

### Nuclear species-specific marker discovery

To develop nuclear species-specific markers using available Expressed Sequence Tag (EST) resources, 41,294 ESTs of TO (GenBank accession numbers: DY802201-DY843494) and 16,441 ESTs of TK (GenBank accession numbers: GO660574-GO672283, DR398435-DR403165) were obtained from NCBI [22, 23] (Collins J, Whalen MC, Nural-Taban AH, Scott D, Hathwaik U, Lazo GR, Cox K, Durant K, Woolsey R, Schegg K, et al. Genomic and proteomic identification of candidates genes and proteins for rubber biosynthesis in *Taraxacum kok-saghyz* (Russian dandelion). 2009. Unpublished; Shintani D. Using EST from *Taraxacum kok-saghyz* root cDNA library to generate candidate rubber biosynthetic genes. 2005. Unpublished). Using the pipeline described by Kozik (2007) [41], ESTs were assembled into contigs and filtered. Interspecific variants were selected manually, by screening alignments flagged as containing interspecific variations.

### Species-specific marker validation

Markers were validated through gel based assays in larger populations than those used for sequencing for each species. The number of genotypes used for TK, TO and TB were 102, 103 and 24, respectively (Additional files 1, 2 and 3). Primers were designed by Primer 3 [42, 43] to validate Cleaved Amplified Polymorphic Sequences (CAPS), which were identified by CAPS Designer [44] using the following PCR procedure: 5 min initial denaturation at 95 °C, followed by 35 cycles of 40s denaturation at 95 °C, 60s annealing at 54 °C or 56 °C, 60s elongation at 68 °C, as well as a final extension step at 68 °C for 5 min. Tetra-primer ARMS-PCR was also carried out to detect SNPs using the similar PCR procedure with a 58 °C annealing temperature [45]. All the PCR reactions were conducted using reagents obtained from New England Biolabs (Inc., Ipswich, MA, USA) in a 10 μL reaction, following the manufacturer’s instructions.

## Results

### Chloroplast genome generation, characterization and annotation

**Fig. 1:**
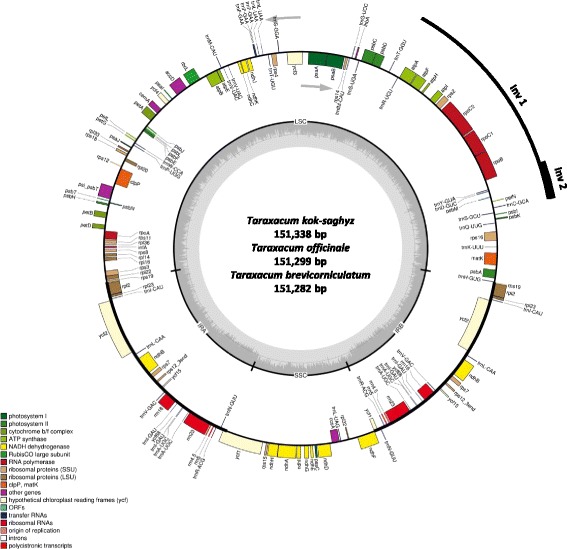
Chloroplast genome annotation map for *Taraxacum kok-saghyz*, *T. officinale* and *T. brevicorniculatum*. Chloroplast genome map represents all three species since their gene number, order and names are the same, except that TO has only two copies of gene *trn*F-GAA. Genes on the outside are transcribed in the counterclockwise direction while genes on the inside are transcribed in the clockwise direction, as shown by the *arrows*. Inv 1 and Inv 2 indicate large and small inversion regions

More than 25 million paired-end reads were generated by the Illumina GAII sequencing platform for TK, while more than 10, 12, and 6 million reads were generated by MiSeq sequencing for multiple genotypes of TK, TO, and TB, respectively. After *de nova* and reference guided assembly, the complete chloroplast genome sequences of TK, TO, and TB were obtained and submitted to NCBI database with GenBank accession numbers KX198560 (TK), KX198561 (TO), and KX198559 (TB). The genome sizes of TK, TO, and TB were found to be 151,338, 151,299, and 151,282 bp, respectively. The genome sizes of these three species are similar to those of other species in the Asteraceae, which range from 149,510 to 153,202 bp [46, 47]. The chloroplast genome can be divided into four regions, which are one Large Single Copy (LSC) region, one Small Single Copy (SSC) region, and two Inverted Repeat (IR) regions. The genome size, and regions, as well as the GC content of each species are listed in Table 1. As previously reported in other Asteraceae, the chloroplast genomes of TK, TO, and TB contain a 21 k large inversion (Inv 1) and a 2.1 k small inversion (Inv 2) in the LSC region (Fig. 1) [19, 48, 49]. Inv 1 begins between gene *trn*S-GCU and *trn*C-GCA, and ends between *trn*R-UCU and *trn*T-GGU. Inv2 occurred at one end of Inv1 and shares the same starting point as Inv1. Inv2 ends between *trn*Y-GUA and *rop*B.

**Table 1:** Chloroplast genomes of *Taraxacum kok-saghyz*, *T. officinale* and *T. brevicorniculatum*

Species	GenBank Accession NO.	Size (bp)				GC%
		Total	SSC	IR	LSC	
*Taraxacum kok-saghyz*	KX198560	151,338	18,472	24,440	83,986	37.7
*Taraxacum officinale*	KX198561	151,299	18,541	24,439	83,880	37.7
*Taraxacum brevicorniculatum*	KX198559	151,282	18,578	24,421	83,862	37.7

The annotated chloroplast genomes of these three species are represented in one circular map since their gene number, order and names are the same (Fig. 1). A total of 134 genes have been identified for each of the three species, including 82 protein-coding genes, 8 rRNA genes, 36 tRNA genes, as well as 8 pseudogenes and Open Reading Frames (ORFs). There are 61 protein-coding genes and 21 tRNA genes located in the LSC region, while 11 protein-coding genes and 1 tRNA gene are located in the SSC region. All the rRNA genes are located in the IR regions, along with 5 protein-coding genes, 7 tRNA genes and 4 pseudogenes and ORFs. Genes located in the IR regions are duplicated except *rps*19 and *ycf*1, which were only partially duplicated. One specific feature of note is that gene *trn*F-GAA has three copies in TK and TB, but only two copies in TO. The copy number variation of the *trn*F-GAA gene has been considered a specific characteristic of *Taraxacum* sp., which might be useful as a resource for evolutionary studies [18, 50].

### Phylogenetic analysis in the Asteraceae

**Fig. 2:**
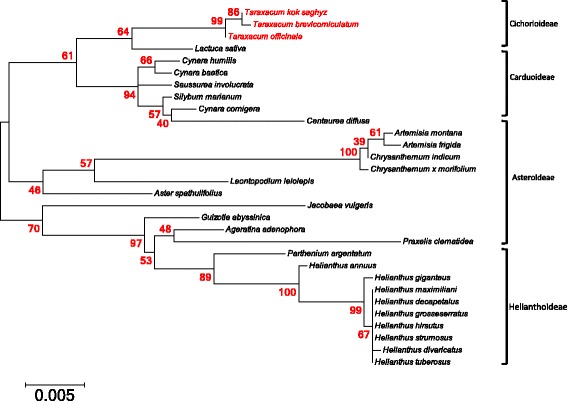
Phylogenetic analysis using *rbc*L gene from 30 species with available chloroplast sequences in the Asteraceae. *Taraxacum kok-saghyz*, *T. brevicorniculatum*, and *T. officinale* are highlighted in *red*. The *bar* at the bottom shows the scale of the branch length representing the number of substitutions per site. Numbers shown next to the nodes indicate the percentage of trees with the associated taxa clustered together

Sequence alignment showed that TK, TO, and TB chloroplast genomes are highly homologous with other members of the Asteraceae. A phylogenetic tree showing the genetic relationship of species in the Asteraceae was obtained (Fig. 2). The results were consistent with previous studies; species within the same subfamily and tribe were grouped together [47, 51]. Phylogenetic analysis showed that TK has a closer genetic distance to TB than to TO. Of the species analyzed, the *Taraxacum* species were most closely related to lettuce (*Lactuca sativa*) (Fig. 2).

### Comparative analysis of chloroplast genomes in *Taraxacum* genus

**Fig. 3:**
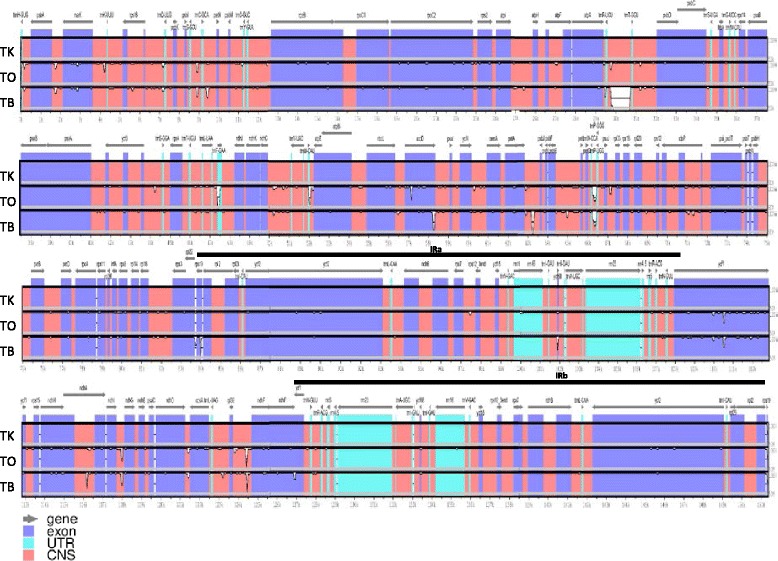
Comparative analysis of *Taraxacum kok-saghyz* (TK), *T. officinale* (TO), and *T. brevicorniculatum* (TB) chloroplast genomes. Comparative analysis was conducted using mVISTA program. Y-axis represents the sequence identity percentage from 50 to 100%. *Grey arrows* indicate gene coding regions with the direction of transcription. *Blue* indicates exons, *green-blue* indicates untranslated regions (UTR), while *pink* indicates conserved non-coding sequences (CNS). *Horizontal black lines* showed the two IR regions

The complete sequences of TK, TO, and TB were compared and revealed specific highly divergent regions (Fig. 3). Overall, the three species were highly similar, with shared sequence identities of 99.6% in pairwise comparisons. Two IR regions are highly conserved among the species. Non-coding regions, including intergenic regions and introns, were more divergent than protein coding regions. Pairwise comparison between species revealed the gene coding regions with most variations. The first 15 regions with the lowest sequence identity are listed in Table 2. Gene *acc*D (*acetyl-CoA carboxylase carboxyltransferase beta subunit*) showed the most divergence among the three species. Gene sequence identities of the 15 regions ranged from 97.97 to 99.79%, 96.34 to 99.81% and 94.38 to 99.79% for TK and TO, TK and TB, TO and TB comparisons, respectively. Additionally, non-coding regions with high divergence include inter spaces between *trn*R-UCU and *trn*T-GGU, *trn*M-CAU and *atp*E, *pet*A and *psb*J, *trn*W-CCA and *trn*P-UGG, *ndh*I and *ndh*G, *rpl*32 and *ndh*F, as well as the intron region of *ndh*A.

**Table 2:** The 15 coding regions with the lowest identity in pairwise comparison of three *Taraxacum* species

TK^a^ vs TO^b^			TK vs TB^c^			TO vs TB		
Coding regions	Length (bp)	Identity (%)	Coding regions	Length (bp)	Identity (%)	Coding regions	Length (bp)	Identity (%)
*acc*D	1530	97.97	*acc*D	1503	96.34	*acc*D	1530	94.38
*pet*L	96	98.96	*pet*L	96	98.96	*psb*F	120	99.17
*ycf*1	5073	99.15	*ycf*1	5073	99.07	*rpl*32	165	99.39
*psb*F	120	99.17	*rpl*32	165	99.39	*ycf*3 exon2	228	99.56
*mat*K	1521	99.41	*mat*K	1521	99.41	*inf*A	234	99.57
*rps*8	405	99.51	*rpl*33	207	99.52	*ycf*1	5067	99.63
*rpl*33	207	99.52	*ycf*3 exon2	228	99.56	*ndh*A exon2	539	99.63
*inf*A	234	99.57	*ndh*I	501	99.60	*rps*14	303	99.67
*ccs*A	969	99.69	*ndh*H	1182	99.66	*ndh*F	2202	99.68
*rps*2	711	99.72	*rps*14	303	99.67	*ccs*A	969	99.69
*rpl*20	381	99.74	*pet*A	963	99.69	*mat*K	1521	99.74
*ndh*H	1182	99.75	*rpl*20	381	99.74	*rps*8	405	99.75
*rps*11	411	99.76	*rps*8	405	99.75	*ndh*H	1182	99.75
*rpl*16	411	99.76	*rps*11	411	99.76	*rpl*16	411	99.76
*pet*A	963	99.79	*ndh*G	531	99.81	*rbc*L	1428	99.79

^a^ *TK Taraxacum kok-saghyz,* ^b^ *TO T. officinale*, ^c^ *TB T. brevicorniculatum*

### Chloroplast species-specific marker development

Variant calling revealed 218 intraspecific variants within 24 genotypes of TK, including 172 SNPs (with an average coverage of 1720, ranging from 46 to 8796), while only 31 intraspecific variants within 24 genotypes of TO were detected, including 12 SNPs (with an average coverage of 7082, ranging from 961 to 24,907). After mapping TO short reads to the TK chloroplast, a total of 281 variants were identified, including 205 SNPs. The average coverage was 1838, and ranged from 152 to 11,954. Among these SNPs, 16 were fixed between TK and TO, with an average coverage of 1708, ranging from 152 to 4296. The location, nucleotide change, annotation and mutation type of these 16 SNPs are listed in Table 3. Although nine SNPs were located in protein coding regions, only two SNPs, which was within gene *acc*D and *ndh*A (*NADH dehydrogenase subunit 1*), caused non-synonymous mutations. Four CAPS markers and one Tetra-primer ARMS-PCR marker were chosen as candidate species-specific markers. The primers and restriction enzymes used for marker detection are included in Table 4. These five markers were further validated in a large TO population, including 24 genotypes used for MiSeq and another 59 genotypes from a TO seed world collection. The markers were also validated in a large TK population, which included multiple genotypes from the USDA TK collection and TK populations from current OSU breeding programs (Additional files 1 and 2). Three CAPS markers and one Tetra-primer ARMS-PCR marker showed fixed band patterns within each species, but these patterns were consistently different between species, suggesting that these markers can be used as species-specific markers to differentiate TK from TO. Combining multiple markers is not required since each single marker is sufficient for species differentiation in our study. Among four CAPS markers tested here, one showed polymorphic band patterns in the TO population and could be used as an intraspecific marker. The band patterns for each marker in each species are listed in Table 4.

**Table 3:** Chloroplast *Taraxacum kok-saghyz* (TK) and *T. officinale* (TO) potential species-specific SNPs

SNP NO.	TK Position	TK SNP	TO Position	TO SNP	Annotation	Mutation type	
1	199	T	199	A	Inter space between *trn*H-GUG and *psb*A	Transversion	-
2	10984	A	10984	C	Inter space between *psb*M and *trn*D-GUC	Transversion	-
3	19580	T	19579	C	*rpo*C2	Transition	Synonymous
4	22844	C	22843	A	*rpo*C2	Transversion	Synonymous
5	55885	T	55798	C	*rbc*L	Transition	Synonymous
6	56829	T	56742	C	Inter space between *rbc*L and *acc*D	Transition	-
7	56954	A	56867	C	*acc*D	Transversion	Synonymous
8	57823	C	57763	G	*acc*D	Transversion	Non-synonymous
9	72351	T	72244	C	*psi_psb*T	Transition	Synonymous
10	73068	G	72961	T	*psi_psb*T	Transversion	Synonymous
11	80173	T	80067	G	*rps*8	Transversion	Synonymous
12	110014	G	109902	A	Inter space between *trn*N-GUU and *rps*15	Transition	-
13	113818	A	113727	C	*ndh*H	Transversion	Synonymous
14	115335	T	115244	C	*ndh*A	Transition	Non-synonymous
15	123069	T	122951	C	Inter space between *trn*L-UAG and *rpl*32	Transition	-
16	135299	C	135261	T	*rrn*16	Transition	-

**Table 4:** Chloroplast *Taraxacum kok-saghyz* (TK) and *T. officinale* (TO) species-specific and intraspecific markers

NO.^a^	Annotation	Forward primer	Reverse primer	Length	Ta	Enzyme	TK	TO
C1	Inter space between *rbc*L and *acc*D	5′-ACTCTTTCCACCCATCCTGT-3′	5′-TGAACCACCATCTTTTCATAGAG-3′	287	54	*Taq*I	Fixed	Fixed
C2	*acc*D	5′-ACTCTTTCCACCCATCCTGT-3′	5′-CGCGATCGGGGTTCTTACTA-3′	671	54	*Nco*I	Fixed	Fixed
C3	*rbc*L	5′-ACCGTTTCTTATTTTGTGCCGA-3′	ACCCTCAGTAGCAAGATCGC	677	54	*Kpn*I	Fixed	Fixed
C4	*rpo*C2	Inner 5′-GAGCACAACCAATCTCTATTCGACCT-3′	Inner 5′-TCCAAGATGTACTCCTACAAGTAAAGTGG-3′	TO:215/411	58		Fixed	Fixed
		Outer 5′-TATTTCTGTAAGTCCTCGAAATGGAATG-3′	Outer 5′-AATTTTATTTTTCCATTAGAAGGGGCTC-3′	TK:251/411				
C5	Inter space between *trn*N-GUU *and rps*15	5′-TCAAAGGATCTATGCGCAATCA-3′	5′-TCGAGAATTGAAGACCCCTAGT-3′	462	54	*Taq*I	Fixed	Polymorphic

^a^C1-3, 5 are CAPS markers and C4 is a Tetra-primer ARMS-PCR marker. C1-4 are species-specific markers and C5 is an intraspecific marker

### Nuclear species-specific marker development

A total of 6187 contigs were assembled from existing TO and TK EST resources, totaling 4.2 Megabases (MB), containing 16,900 redundantly detected variants. Only variants detected more than once were counted to reduce noise caused by sequencing errors. A total of 23 redundant, putatively species-specific SNPs were tested as CAPS in the TK and TO populations mentioned above. Of these, two (9%) did not exhibit diversity, 16 (69%) were polymorphic within either or both species and five (22%) were fixed between TK and TO (Table 5). Although some of the five fixed markers showed polymorphic patterns within species, they still differed between the two species, and so were considered species-specific markers. Species-specific markers fixed between TK and TO, as well as intraspecific markers fixed in parental populations, can be further used to validate potential TK x TO hybrid populations in gene flow studies.

**Table 5:** Nuclear *Taraxacum kok-saghyz* (TK) and *T. officinale* (TO) species-specific and intraspecific markers

NO.^a^	Annotation	Forward primer	Reverse primer	Length	Ta	Enzyme	TK	TO
N1	Tubulin alpha-2 alpha-4 chain	5′-ATGGTCAGATGCCCAGTGA-3′	5′-TGTCGTAGATGGCTTCGTTG-3′	540	56	*Hinf*I	Polymorphic	Polymorphic
N2	Tubulin alpha-2 alpha-4 chain	5′-GATTTGGTGAACAATTTGGGTA-3′	5′-TCATCATCGGAGATTTCTTTCTC-3′	401	54	*Msp*I	Fixed	Fixed
N3	Subtilase family protein	5′-TGGATTTTTATGCACGACACC-3′	5′-CCGCACCTTATGCCCTCT-3′	358	56	*Msp*I	Fixed	Fixed
N4	Tubulin alpha-2 alpha-4 chain	5′-ATGGTCAGATGCCCAGTGA-3′	5′-TGTCGTAGATGGCTTCGTTG-3′	540	56	*Alu*I	Polymorphic	Polymorphic
N5	Tetraspanin family protein	5′-AGGGGTCTTGATCTTGGTTG-3′	5′-CTTGAGCCATGCGGTAAGTT-3′	323	54	*Dpn*II	Fixed	Polymorphic
N6	Subtilase family protein	5′-TGGATTTTTATGCACGACACC-3′	5′-CCGCACCTTATGCCCTCT-3′	358	56	*Alu*I	Polymorphic	Polymorphic
N7	Tetraspanin family protein	5′-AGGGGTCTTGATCTTGGTTG-3′	5′-CTTGAGCCATGCGGTAAGTT-3′	323	54	*Rsa*I	Fixed	Polymorphic
N8	NAC domain-containing protein 2	5′-ATGAGTACCGCCTCGCTAAC-3′	5′-GCTTCGCTTTGAACTTCTCC-3′	343	54	*Hinf*I	Polymorphic	Polymorphic
N9	Aquaporin tip2-2	5′-TGGAGATCATCATCACATTTGC-3′	5′-GGGTAAATGAGACCAGCTAGACC-3′	265	56	*Ban*I	Polymorphic	Polymorphic
N10	Aquaporin pip1-1	5′-CTCGGAGCCAACAAGTTTTC-3′	5′-CAGCGGTGCAGTAGACAAGA-3′	295	56	*Hinf*I	Polymorphic	Polymorphic
N11	Aquaporin pip1-1	5′-CTCGGAGCCAACAAGTTTTC-3′	5′-CAGCGGTGCAGTAGACAAGA-3′	295	56	*Msp*I	Polymorphic	Fixed
N12	Enoyl reductase	5′-ACTACTCGGAGCGGAAGAGA-3′	5′-AATCACCCCAAACCCTAACC-3′	606	54	*Hinf*I	Fixed	Polymorphic
N13	Enoyl reductase	5′-ACTACTCGGAGCGGAAGAGA-3′	5′-AATCACCCCAAACCCTAACC-3′	606	54	*Mlu*I	Fixed	Polymorphic
N14	Cinnamyl alcohol dehydrogenase 5	5′-TGATGTTTACACCGACGGTAA-3′	5′-AGCATGAGGAGAGGGGAGAC-3′	504	54	*Hae*III	Polymorphic	Fixed
N15	Cinnamyl alcohol dehydrogenase 5	5′-TGATGTTTACACCGACGGTAA-3′	5′-AGCATGAGGAGAGGGGAGAC-3′	504	54	*Mlu*I	Polymorphic	Fixed
N16	Cinnamyl alcohol dehydrogenase 5	5′-TGATGTTTACACCGACGGTAA-3′	5′-AGCATGAGGAGAGGGGAGAC-3′	504	54	*Scrf*I	Polymorphic	Fixed
N17	Subtilase family protein	5′-TGGATTTTTATGCACGACACC-3′	5′-CCGCACCTTATGCCCTCT-3′	358	56	*Dpn*II	Polymorphic	Polymorphic
N18	Subtilase family protein	5′-TGGATTTTTATGCACGACACC-3′	5′-CCGCACCTTATGCCCTCT-3′	358	56	*BSTE*II	Polymorphic	Fixed
N19	Subtilase family protein	5′-TGGATTTTTATGCACGACACC-3′	5′-CCGCACCTTATGCCCTCT-3′	358	56	*Xba*l	Polymorphic	Fixed
N20	Aquaporin tip2-2	5′-TGGAGATCATCATCACATTTGC-3′	5′-GGGTAAATGAGACCAGCTAGACC-3′	265	56	*Alu*I	Fixed	Polymorphic
N21	Aquaporin tip2-2	5′-TGGAGATCATCATCACATTTGC-3′	5′-GGGTAAATGAGACCAGCTAGACC-3′	265	56	*Scrf*I	Fixed	Polymorphic

^a^N1-5 are species-specific markers and N6-21 are intraspecific markers

## Discussion

**Fig. 4:**
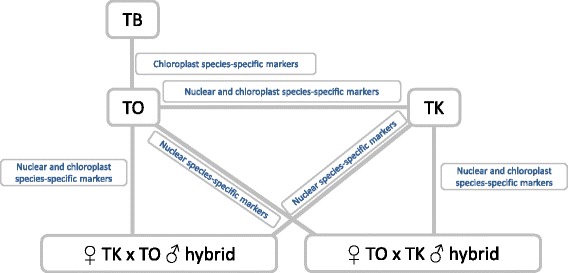
Relationship between *Taraxacum* species-specific markers and their functions in species differentiation studies. Chloroplast species-specific markers of *Taraxacum kok-saghyz* (TK), *T. officinale* (TO) and *T. brevicorniculatum* (TB) are inherited maternally, which can be used to differentiate TB and TK from TO, as well as track the maternal ancestors of potential ♀ TK x TO ♂ and ♀ TO x TK ♂ hybrids in the absence of parental information. Nuclear species-specific markers can be used to differentiate TK from TO, as well as TK and TO from their hybrid progeny

In this study, we generated chloroplast genomes for three *Taraxacum* species using Illumina GAII and MiSeq platforms, which provide important resources for ecological, evolutionary, and genetic engineering studies. The TO chloroplast genome was compared to previously published data and showed highly similarity in genome size and structure, gene number, as well as GC content [51]. Using chloroplast sequences obtained here, and online EST data, we were able to develop species-specific and intraspecific molecular markers, which are essential tools for germplasm purification and gene flow studies. The relationship between different types of molecular markers and species differentiation are summarized in Fig. 4. In our study, we developed new strategies to conduct chloroplast sequencing to facilitate species-specific marker discovery. By pooling multiple genotypes with normalized molarity into one library, and sequencing three libraries in one MiSeq run, a wide range of variation was detected at low cost. After SNP calling, we further validated the candidate markers using populations containing a wide germplasm collection. Not all the candidate markers revealed by sequencing were confirmed as species-specific markers, indicating that only using sequencing data from limited genotypes is not sufficient to develop reliable species-specific markers. Gel-based assays used in this study allowed us to develop species-specific markers which can be used through broad populations, which is especially critical for outcrossing species (e.g. TK) and species with widespread geographic distribution (e.g. TO).

It is expected that the most likely avenue of hybridization between TK and TO is the pollination of TK by TO, as the majority of TO are obligate apomicts [10, 11]. TK was introduced into the U.S. during the Emergency Rubber Project in 1942, where it was hastily cultivated at 152 locations in 40 states as a source of rubber [5]. Although the project was abandoned in 1944 [5], the massive introduction of TK gave considerable opportunity for gene flow between TK and its weedy relative TO. Although no TK plants appear to have persisted, no gene flow risk assessment between these two species has been reported. Maternally inherited chloroplast species-specific markers provided in this study can be used to detect ancestral hybridization in the field between TK mothers and TO fathers, even when many decades of potential backcrosses may have masked the TK phenotype in such hybrids. The recent development of TK as an alternative rubber resource has prompted new germplasm introductions from Uzbekistan and Kazakhstan in 2006 and 2009, respectively. These markers can also be used to proactively detect recent hybridizations, which may now be occurring.

In this study, intraspecific chloroplast and nuclear markers have been discovered, which may have uses in population genetics to test correlations between genetic information carried by chloroplast and nuclear genomes and geographic or environmental data. Intraspecific markers can be used to characterize population structures, revealing information about local adaptation, important evolutionary events and genetic communication frequencies. Additionally, intraspecific nuclear markers also can be used to validate hybrids in controlled crosses, develop genetic maps and conduct marker assisted breeding.

When the chloroplast sequences of TK, TO, and TB were compared, it became apparent that TK and TB were highly similar. Moreover, all four gel-based markers that could discriminate TK and TO could not distinguish TK from TB. These results suggested that TB and TK might share a maternal ancestor. This result supports the finding that the triploid genome of TB is composed of two copies of the TK genome with one copy of an unknown dandelion species (personal communication with Dr. J. Kirschner, Institute of Botany, Academy of Sciences, 25243, Průhonice 1, Czech Republic, 2010). This study may enable additional research on *Taraxacum* chloroplast diversity, by demonstrating a complement of primers that can amplify entire chloroplast genomes. Furthermore, it may inform chloroplast sequencing efforts to resolve *Taraxacum* phylogenies, by revealing which regions may have higher interspecific diversity.

The complete annotated chloroplast sequences for TK, TO, and TB allows the development of chloroplast engineering within *Taraxacum*. The availability of the native chloroplast sequences of an organism can allow constructs to be designed to more readily achieve homologous recombination [52]. Chloroplast engineering is a powerful tool that provides a high level of transgene expression because of the polyploid nature of chloroplast genomes and the large number of chloroplasts present in a single plant cell [53]. Furthermore, chloroplast engineering should prevent the escape of transgenes via pollen, as chloroplasts are maternally inherited [54, 55]. Chloroplast engineering also allows multigene transformation and chloroplast gene manipulation [56]. This research may enable chloroplast engineering in TK and TB to divert additional assimilate to rubber production [57].

## Conclusions

The chloroplast sequences obtained from multiple genotypes within each of the three dandelion species investigated, along with online EST data, allowed us to develop species-specific and intraspecific molecular markers. The availability of chloroplast genomes of these three dandelion species, as well as chloroplast and nuclear molecular markers, provides a powerful genetic resource for germplasm differentiation and purification, and the study of potential gene flow among *Taraxacum* species. This will further facilitate ecological, evolutionary, and genetic engineering studies for these three species, and significantly accelerate the development of TK as a domestic rubber-producing crop.

## Additional files

Additional file 1:
*Taraxacum kok-saghyz* genotypes for sequencing and marker validation. (DOCX 28 kb)
Additional file 2:
*Taraxacum officinale* genotypes for sequencing and marker validation. (DOCX 47 kb)
Additional file 3:
*Taraxacum brevicorniculatum* genotypes for sequencing and marker validation. (DOCX 28 kb)
Additional file 4: Primers used for chloroplast genome amplification by Long Range PCR. (DOCX 27 kb)
Additional file 5: Rubisco large subunit genes (*rbc*L) from the Asteraceae. (DOCX 29 kb)

## Abbreviations

AFLP: Amplified fragment length polymorphism
CAPS: Cleaved amplified polymorphic sequences
CTAB: Cetyl trimethylammonium bromide
DOGMA: Dual Organellar GenoMe Annotator
EST: Expressed sequence tag
IR: Inverted repeat
LSC: Large single copy
MB: Megabase
MCIC: The molecular and cellular imaging center
NCBI: National Center for Biotechnology Information
OARDC: Ohio Agricultural Research and Development Center
OGDRAW: OrganellarGenomeDRAW
ORF: Open reading frame
PCR: Polymerase chain reaction
SNP: Single nucleotide polymorphism
SSC: Small single copy
TB: *Taraxacum brevicorniculatum*
TK: *Taraxacum kok-saghyz*
TO: *Taraxacum officinale*
