# Supplementary material for: Chloroplast genome resources and molecular markers differentiate rubber dandelion species from weedy relatives
Source: BMC Plant Biol. 2017 Feb 2;17:34. doi: 10.1186/s12870-016-0967-1 (PMC5289045; doi:10.1186/s12870-016-0967-1)
Supplement: Additional file 1: — Taraxacum kok-saghyz genotypes for sequencing and marker validation. (DOCX 28 kb) [file 12870_2016_967_MOESM1_ESM.docx]

**Additional file 1** *Taraxacum kok-saghyz* genotypes for sequencing and marker validation

| USDA Accession NO. | ACP | ACNO | NO. of Plants Used for MiSeq | NO. of Plants Used for Marker Validation |
| --- | --- | --- | --- | --- |
| KAZ08-001 | W6 | 35156 | 1 | 2 |
| KAZ08-004 | W6 | 35159 | 1 | 4 |
| KAZ08-005 | W6 | 35160 | 1 | 4 |
| KAZ08-007 | W6 | 35162 | 1 | 2 |
| KAZ08-009 | W6 | 35164 | 1 | 2 |
| KAZ08-010 | W6 | 35165 | 0 | 3 |
| KAZ08-011 | W6 | 35166 | 1 | 4 |
| KAZ08-013 | W6 | 35168 | 1 | 5 |
| KAZ08-014 | W6 | 35169 | 1 | 6 |
| KAZ08-015 | W6 | 35170 | 1 | 10 |
| KAZ08-017 | W6 | 35172 | 1 | 6 |
| KAZ08-018 | W6 | 35173 | 1 | 3 |
| KAZ08-021 | W6 | 35176 | 1 | 5 |
| KAZ08-022 | W6 | 35177 | 1 | 3 |
| KAZ08-023 | W6 | 35178 | 1 | 2 |
| KAZ08-024 | W6 | 35179 | 1 | 2 |
| KAZ08-025 | W6 | 35180 | 1 | 2 |
| KAZ08-026 | W6 | 35181 | 1 | 3 |
| KAZ08-027 | W6 | 35182 | 1 | 2 |
| KAZ08-028 | W6 | 35183 | 1 | 2 |
| Cross 1×4 | - | - | 1 | 1 |
| Cross 9×11 | - | - | 1 | 1 |
| Cross 10×14 | - | - | 1 | 1 |
| Cross 22×23 | - | - | 1 | 1 |
| Male sterile 13 | - | - | 1 | 2 |
| Breeding population | - | - | 0 | 24 |
| Total |  |  | 24 | 102 |
