# Supplementary material for: Chloroplast genome resources and molecular markers differentiate rubber dandelion species from weedy relatives
Source: BMC Plant Biol. 2017 Feb 2;17:34. doi: 10.1186/s12870-016-0967-1 (PMC5289045; doi:10.1186/s12870-016-0967-1)
Supplement: Additional file 2: — Taraxacum officinale genotypes for sequencing and marker validation. (DOCX 47 kb) [file 12870_2016_967_MOESM2_ESM.docx]

**Additional file 2** *Taraxacum officinale* genotypes for sequencing and marker validation

| Source NO.^1^ | Specimen NO. | City | State/Province | Country | Zip | NO. of Plants Used for MiSeq | NO. of Plants Used for Marker Validation | |
| --- | --- | --- | --- | --- | --- | --- | --- | --- |
| 1 | **1** | Ashville | NC | USA | 28805 | 1 | 2 |  |
| 3 | **1** | Westmoreland | KS | USA | 66549 | 0 | 1 |  |
| 6 | **6** | Amherst | MA | USA | 01002 | 1 | 1 |  |
| 7 | **3** | Davis | CA | USA | 95616 | 1 | 1 |  |
| 12 | **1** | Delmar | DE | USA | 19940 | 1 | 2 |  |
| 15 | **1** | Madison | WI | USA | 53703 | 0 | 1 |  |
| 22 | 2 | Troy | OH | USA | 45373 | 0 | 1 |  |
| 22 | 3 | Ludlow Falls | OH | USA | 45339 | 0 | 1 |  |
| 22 | 4 | Covington | OH | USA | 45318 | 0 | 1 |  |
| 22 | **5** | Tipp City | OH | USA | 45371 | 0 | 1 |  |
| 22 | **7** | Troy | OH | USA | 45373 | 0 | 1 |  |
| 24 | **1** | Ames | IA | USA | 50014 | 1 | 1 |  |
| 25 | **4** | Westlake | OH | USA | 44145 | 0 | 1 |  |
| 27 | **1** | Indianapolis | IN | USA | 46208 | 1 | 1 |  |
| 29 | **4** | Indianapolis | IN | USA | 46268 | 0 | 1 |  |
| 31 | **1** | Corvallis | OR | USA | 97330 | 1 | 1 |  |
| 36 | **1** | East Lansing | MI | USA | 48823 | 0 | 1 |  |
| 38 | **1** | Wilmette | IL | USA | 60091 | 0 | 1 |  |
| 43 | **4** | Richmond | VA | USA | 23233 | 0 | 1 |  |
| 45 | **4** | Lakewood | OH | USA | 44107 | 0 | 1 |  |
| 45 | **5** | North Olmsted | OH | USA | 44070 | 0 | 1 |  |
| 45 | **6** | North Olmsted | OH | USA | 44070 | 0 | 1 |  |
| 45 | **7** | North Olmsted | OH | USA | 44070 | 0 | 1 |  |
| 45 | **8** | North Olmsted | OH | USA | 44070 | 0 | 1 |  |
| 50 | **1** | Lexington | KY | USA | 40509 | 0 | 1 |  |
| 51 | **1** | Darien | IL | USA | 60561 | 0 | 1 |  |
| 54 | **3** | Columbus | OH | USA | 43235 | 0 | 1 |  |
| 55 | **6** | Alabaster | AL | USA | 35007 | 0 | 1 |  |
| 60 | **1** | Pennsylvania Furnace | PA | USA | 16865 | 1 | 1 |  |
| 62 | **4** | State College | PA | USA | 16803 | 0 | 1 |  |
| 64 | **4** | Furnace | PA | USA | 16865 | 0 | 1 |  |
| 65 | **1** | State College | PA | USA | 16803 | 0 | 1 |  |
| 68 | **1** | Pine Grove Mills | PA | USA | 16868 | 0 | 1 |  |
| 69 | 1 | Marcellus | NY | USA | 13108 | 1 | 1 |  |
| 73 | 2 | Belington | WV | USA | 26250 | 0 | 1 |  |
| 75 | **3** | Bluff City | TN | USA | 37618 | 0 | 1 |  |
| 76 | **1** | Red House | WV | USA | 25168 | 1 | 1 |  |
| 76 | **3** | Red House | WV | USA | 25168 | 0 | 1 |  |
| 77 | **4** | Anderson | SC | USA | 29625 | 0 | 1  Continued |  |
| 81 | **2** | Ft. Campbell | KY | USA | 42223 | 0 | 1 |  |
| 82 | **3** | Clemson | SC | USA | 29631 | 0 | 1 |  |
| 83 | **4** | Tulsa | OK | USA | 74136 | 0 | 1 |  |
| 91 | **1** | Woodstock | Ontario | Canada | N4S 7V7 | 1 | 1 |  |
| 92 | **4** | Croton-On-Hudson | NY | USA | 10520 | 0 | 1 |  |
| 100 | **2** | Lexington | KY | USA | 40511 | 0 | 1 |  |
| 101 | **3** | Elm City | NC | USA | 27822 | 0 | 1 |  |
| 103 | **1** | Salt Lake City | UT | USA | 84105 | 0 | 1 |  |
| 107 | **2** | St. Louis | MO | USA | 63141 | 0 | 1 |  |
| 108 | **1** | Webster Groves | MO | USA | 63119 | 0 | 1 |  |
| 109 | **4** | Labadie | MO | USA | 63055 | 0 | 1 |  |
| 110 | **4** | Oxford | OH | USA | 45056 | 0 | 1 |  |
| 111 | **1** | Laurel | IN | USA | 47024 | 0 | 1 |  |
| 114 | **4** | Maplewood | MN | USA | 55109 | 0 | 1 |  |
| 116 | **1** | Vivan | SD | USA | 57576 | 0 | 1 |  |
| 116 | **13** | Hancock | MI | USA | 49930 | 0 | 1 |  |
| 117 | **1** | Max Meadow | VA | USA | 24360 | 0 | 1 |  |
| 123 | **1** | Sault Ste. Marie | ON | Canada | P6A 6K4 | 0 | 1 |  |
| 124 | **1** | Munich | Bavaria Land | Germany | 81927 | 0 | 1 |  |
| 125 | **1** | Clifton | VA | USA | 20124 | 0 | 1 |  |
| 128 | **1** | Auburn University | AL | USA | 36849 | 0 | 1 |  |
| 130 | **8** | Pembenville | OH | USA | 43450 | 1 | 1 |  |
| 130 | **12** | New Carlisle | OH | USA | 45344 | 0 | 1 |  |
| 131 | **4** | Starkville | MS | USA | 39759 | 0 | 1 |  |
| 132 | **1** | Vizzola Ticino | Varese | Italy | 21010 | 0 | 2 |  |
| 133 | **1** | Frankfort | KY | USA | 40601 | 0 | 1 |  |
| 135 | **4** | Bristow | OK | USA | 74010 | 0 | 1 |  |
| 138 | **1** | Dürnstein | Lower Austria | Austria | 3601 | 1 | 2 |  |
| 138 | **3** | Lyoblana | / | Slovenia | 1000 | 1 | 1 |  |
| 140 | **1** | Shreve | OH | USA | 44676 | 1 | 1 |  |
| 146 | **16** | Kelso | ND | USA | 58045 | 0 | 1 |  |
| 147 | **2** | Shenyang | Liaoning | China | 110000 | 0 | 8 |  |
| 147 | **7** | Shenyang | Liaoning | China | 110000 | 1 | 1 |  |
| 148 | **1** | Wageningen | Gelderland | Netherlands | 6700 | 0 | 11 |  |
| 151 | **13** | Athens | OH | USA | 45701 | 1 | 1 |  |
| / | **OH2** | Wooster | OH | USA | 44691 | 0 | 1 |  |
| / | **IA2** | Ames | IA | USA | 50010 | 1 | 1 |  |
| / | **IA3** | Ames | IA | USA | 50010 | 1 | 1 |  |
| / | **MN3** | / | MN | USA | / | 1 | 1 |  |
| / | **NY1** | Marcellus | NY | USA | 13108 | 1 | 1 |  |
| / | **OH1** | Columbus | OH | USA | 43215 | 1 | 1  Continued |  |
| / | **PA2** | Lancaster | PA | USA | 17602 | 1 | 1 |  |
| / | **PA3** | Lancaster | PA | USA | 17602 | 1 | 1 |  |
| Total |  |  |  |  |  | 24 | 103 |  |

^1^ Source NO. indicates the collector of *Taraxacum officinale* seed.
