# Supplementary material for: Chloroplast genome resources and molecular markers differentiate rubber dandelion species from weedy relatives
Source: BMC Plant Biol. 2017 Feb 2;17:34. doi: 10.1186/s12870-016-0967-1 (PMC5289045; doi:10.1186/s12870-016-0967-1)
Supplement: Additional file 3: — Taraxacum brevicorniculatum genotypes for sequencing and marker validation. (DOCX 28 kb) [file 12870_2016_967_MOESM3_ESM.docx]

**Additional file 3** *Taraxacum brevicorniculatum* genotypes for sequencing and marker validation

| Genotype | Source | NO. of Plants Used for MiSeq | NO. of Plants Used for Marker Validation |
| --- | --- | --- | --- |
| *T. brevicorniculatum*,  clone A | Botanical garden, Marburg University, Germany | 1 | 3 |
| Line 2 | Kazakhstan collection | 0 | 1 |
| Line 5 | Kazakhstan collection | 0 | 1 |
| Line 8 | Kazakhstan collection | 0 | 1 |
| Line 9 | Kazakhstan collection | 1 | 1 |
| Line 10 | Kazakhstan collection | 1 | 0 |
| Line 12 | Kazakhstan collection | 0 | 1 |
| Line 14 | Kazakhstan collection | 1 | 1 |
| Line 15 | Kazakhstan collection | 1 | 1 |
| Line 17 | Kazakhstan collection | 1 | 1 |
| Line 18 | Kazakhstan collection | 0 | 1 |
| Line 20 | Kazakhstan collection | 1 | 1 |
| Line 21 | Kazakhstan collection | 1 | 1 |
| Line 22 | Kazakhstan collection | 1 | 1 |
| Line 24 | Kazakhstan collection | 0 | 1 |
| Line 28 | Kazakhstan collection | 0 | 1 |
| Line 29 | Kazakhstan collection | 0 | 1 |
| Line 30 | Kazakhstan collection | 0 | 1 |
| Line 32 | Kazakhstan collection | 0 | 1 |
| Line 33 | Kazakhstan collection | 0 | 1 |
| Line 35 | Kazakhstan collection | 0 | 1 |
| Line 37 | Kazakhstan collection | 1 | 0 |
| Line 38 | Kazakhstan collection | 0 | 1 |
| Line 39 | Kazakhstan collection | 1 | 1 |
| Total |  | 12 | 24 |

Note: Kazakhstan collection – collected by Dr. Anvar Buranov, Delta Plant Technologies
