# Supplementary material for: Chloroplast genome resources and molecular markers differentiate rubber dandelion species from weedy relatives
Source: BMC Plant Biol. 2017 Feb 2;17:34. doi: 10.1186/s12870-016-0967-1 (PMC5289045; doi:10.1186/s12870-016-0967-1)
Supplement: Additional file 4: — Primers used for chloroplast genome amplification by Long Range PCR. (DOCX 27 kb) [file 12870_2016_967_MOESM4_ESM.docx]

**Additional file 4** Primers used for chloroplast genome amplification by Long Range PCR

| Primer Set NO. | | Forward Primer Sequence | Reverse Primer Sequence |
| --- | --- | --- | --- |
| TKcp contig1 | 5’-CTCCCAAGCGCACGAATTTT-3’ | | 5’-AAGTTCTTCTGTCAAACCCTGA-3’ |
| TKcp contig2 | 5’-TGACGACAGGAGGGCAATAA-3’ | | 5’-GAGTCCTTCGCGTAAATTGCT-3’ |
| TKcp contig3 | 5’-ACTCAGCGCAATATGGAGGT-3’ | | 5’-TTGATTGGGCCGAATCGTTG-3’ |
| TKcp contig4 | 5’-TCCCTGTGATGTTCCTTGGA-3’ | | 5’-CGGGGTAGAGCAGTTTGGTA-3’ |
| TKcp contig5 | 5’-TCCAAAACCGAAATGACCCC-3’ | | 5’-TAGATCCGAACACTTGCCCC-3’ |
| TKcp contig6 | 5’-AACGAGCACCGTGGAAATAC-3’ | | 5’-GCAAATGCCTGAACCAAAAT-3’ |
| TKcp contig7 | 5’-TCCTTCGGATTGAGCTGACA-3’ | | 5’-ATCACGCTCCTATTGCCACA-3’ |
| TKcp contig8 | 5’-TTCATACGGCGGGAGTCATT-3’ | | 5’-ACAGGATGGGTGGAAAGAGT-3’ |
| TKcp contig9 | 5’-GACGCGATCTTGCTACTGAG-3’ | | 5’-GCCTAGCTGTACCTACCGTT-3’ |
| TKcp contig10 | 5’-AGAACTCAACGGGACCTTCT-3’ | | 5’-TGCTTCCATAGATTCGATCGTG-3’ |
| TKcp contig11 | 5’-CTACGGCGGTGAACTCAATG-3’ | | 5’-AGGGCCCCAATTGGTAGAAA-3’ |
| TKcp contig12 | 5’-ATGCCCGAGACCAGGTTATT-3’ | | 5’-GAAAACGTCCGGGTCGAAAT-3’ |
| TKcp contig13 | 5’-CGAAGAGAAGCAAATGAAAGGC-3’ | | 5’-TGTGACCCATTATCCAACCAAG-3’ |
| TKcp contig14 | 5’-TGTACCAGTTATCGTGCCCA-3’ | | 5’-GGAAACGCTCGGATGTGATC-3’ |
| TKcp contig15 | 5’-CCGTACATATGGGTTCCCGA-3’ | | 5’-TTAAAACAGGATTCGCGCCC-3’ |
| TKcp contig16 | 5’-CGGACGAATCCACTTTGAAT-3’ | | 5’-GGCGTTAGAGCATTGAGAGG-3’ |
| TKcp contig17 | 5’-GGTAGGAGGTGGGCGTATTT-3’ | | 5’-TTCTGTCTTGGTCCGAGTGG-3’ |
| TKcp contig18 | 5’-TGTAGCTCCAGAATGTTTCAGT-3’ | | 5’-AGATGTATACAGAGGGAAAGCC-3’ |
