# Supplementary material for: Chloroplast genome resources and molecular markers differentiate rubber dandelion species from weedy relatives
Source: BMC Plant Biol. 2017 Feb 2;17:34. doi: 10.1186/s12870-016-0967-1 (PMC5289045; doi:10.1186/s12870-016-0967-1)
Supplement: Additional file 5: — Rubisco large subunit genes (rbcL) from the Asteraceae. (DOCX 29 kb) [file 12870_2016_967_MOESM5_ESM.docx]

**Additional file 5** Rubisco large subunit genes (*rbc*L) from the Asteraceae

| **Tax ID** | **Organism Name** | **Gene ID** | **Accession NO.** | **Start position** | **End Position** | **Length** |
| --- | --- | --- | --- | --- | --- | --- |
| 176616 | *Ageratina adenophora* | 10744244 | NC_015621.1 | 54798 | 56231 | 1434 |
| 395280 | *Artemisia frigida* | 14841283 | NC_020607.1 | 54744 | 56201 | 1458 |
| 669134 | *Artemisia montana* | 22549548 | NC_025910.1 | 54755 | 56212 | 1458 |
| 947974 | *Aster spathulifolius* | 25021341 | NC_027434.1 | 53664 | 55121 | 1458 |
| 124929 | *Centaurea diffusa* | 19592101 | NC_024286.1 | 54608 | 56041 | 1434 |
| 146995 | *Chrysanthemum indicum* | 14657614 | NC_020320.1 | 54765 | 56222 | 1458 |
| 41568 | *Chrysanthemum* x *morifolium* | 14445015 | NC_020092.1 | 54744 | 56201 | 1458 |
| 293558 | *Cynara baetica* | 26042637 | NC_028005.1 | 54677 | 56110 | 1434 |
| 157702 | *Cynara cornigera* | 26042774 | NC_028006.1 | 54633 | 56066 | 1434 |
| 41561 | *Cynara humilis* | 24288108 | NC_027113.1 | 54685 | 56118 | 1434 |
| 4230 | *Guizotia abyssinica* | 6219168 | NC_010601.1 | 54598 | 56055 | 1458 |
| 4232 | *Helianthus annuus* | 4055709 | NC_007977.1 | 54553 | 56010 | 1458 |
| 73283 | *Helianthus decapetalus* | 17962283 | NC_023110.1 | 54590 | 56047 | 1458 |
| 73285 | *Helianthus divaricatus* | 17962150 | NC_023109.1 | 54602 | 56059 | 1458 |
| 73288 | *Helianthus giganteus* | 17961884 | NC_023107.1 | 54599 | 56056 | 1458 |
| 73291 | *Helianthus grosseserratus* | 17962017 | NC_023108.1 | 54599 | 56056 | 1458 |
| 73293 | *Helianthus hirsutus* | 17962416 | NC_023111.1 | 54596 | 56053 | 1458 |
| 73297 | *Helianthus maximiliani* | 17962815 | NC_023114.1 | 54601 | 56058 | 1458 |
| 382522 | *Helianthus strumosus* | 17962682 | NC_023113.1 | 54595 | 56052 | 1458 |
| 4233 | *Helianthus tuberosus* | 17962549 | NC_023112.1 | 54598 | 56055 | 1458 |
| 98722 | *Jacobaea vulgaris* | 10610182 | NC_015543.1 | 54373 | 55830 | 1458 |
| 4236 | *Lactuca sativa* | 3772894 | NC_007578.1 | 55064 | 56497 | 1434 |
| 1582844 | *Leontopodium leiolepis* | 25769264 | NC_027835.1 | 54580 | 56037 | 1458 |
| 35935 | *Parthenium argentatum* | 8655821 | NC_013553.1 | 55303 | 56760 | 1458 |
| 287011 | *Praxelis clematidea* | 18887581 | NC_023833.1 | 54970 | 56427 | 1458 |
| 92921 | *Silybum marianum* | 26043894 | NC_028027.1 | 55209 | 56642 | 1434 |
| 200489 | *Saussurea involucrata* | 26897423 | NC_029465.1 | 1 | 1428 | 1428 |
